# Impact of leukoaraiosis or blood pressure on clinical outcome, mortality and symptomatic intracerebral hemorrhage after mechanical thrombectomy in acute ischemic stroke

**DOI:** 10.1038/s41598-022-25171-9

**Published:** 2022-12-16

**Authors:** Annika Göthel-Ezzeiani, Olav Jansen, Friederike Austein, Amelie Hofmann, Daniela Berg, Johannes Meyne, Johannes Hensler

**Affiliations:** 1 Department of Internal Medicine I, University Hospital Schleswig-Holstein, Campus Kiel, Christian-Albrechts University Kiel, Kiel, Germany; 2 Department of Radiology and Neuroradiology, University Hospital Schleswig-Holstein, Campus Kiel, Christian-Albrechts University Kiel, Kiel, Germany; 3 Department of Neurology, University Hospital Schleswig-Holstein, Campus Kiel, Christian-Albrechts University Kiel, Kiel, Germany; 4 Department of Diagnostic and Interventional Neuroradiology, University Medical Center Hamburg-Eppendorf, Hamburg, Germany; 5 Department of Anesthesia, Friedrich-Ebert-Krankenhaus Neumünster, Neumünster, Germany

**Keywords:** Stroke, Risk factors, Brain imaging, Disability, Comorbidities

## Abstract

We aimed to study the impact of leukoaraiosis (LA) and blood pressure (BP) on clinical outcome, mortality and symptomatic intracerebral hemorrhage (sICH) in acute ischemic stroke (AIS) patients treated with mechanical thrombectomy (MT). We analyzed data retrospectively from 521 patients with anterior large vessel occlusion treated with MT. LA was dichotomized in 0–2 (absent-to-moderate) versus 3–4 (moderate-to-severe) according to the van Swieten scale. Various systolic (SBP) and diastolic (DBP) BP parameters during the first 24 h were collected. Multivariable logistic regressions were performed to identify predictors of a poor 90-day outcome, mortality and sICH. LA was significantly associated with poor outcome (OR 3.2; p < 0.001) and mortality (OR 3.19; p = 0.008), but not sICH (p = 0.19). Higher maximum SBP was significantly associated with poor outcome (OR per 10 mmHg increase = 1.21; p = 0.009) and lower mean DBP was a predictor of mortality (OR per 10 mmHg increase = 0.53; p < 0.001). In the univariate analysis high SBP variability was associated with poor outcome, mortality and sICH, but not in the multivariate model. There was no association between BP and sICH. Severity of LA, SBP variability, high maximum SBP and low DBP are associated with either poor outcome or mortality in AIS patients undergoing MT. However, neither LA nor BP were associated with sICH in our cohort. Thus, mechanisms of the negative impact on outcome remain unclear. Further studies on impact of BP course and its mechanisms and interventions are needed to improve outcome in patients undergoing MT.

## Introduction

Mechanical thrombectomy (MT) has been standard therapy for acute ischemic stroke (AIS) caused by large vessel occlusion (LVO) since publication of the randomized controlled trials in 2015^1^. Although results after MT exceed those after intravenous thrombolysis (IVT), still 55% of patients remain disabled or die within 3 months after stroke^2^. Improvement of prognosis needs knowledge of factors that may influence the effect of treatment.

LA is a radiological result seen as abnormal hypodensity in computed tomography (CT) or T2w-hyperintensity in magnetic resonance imaging (MRI)^3^. It is an expression of microangiopathic damage caused by different reasons as i.e. aging and pre-existing hypertension^4^. The presence of LA has been identified as a negative prognostic marker in many situations like increased risk of dementia, prognosis in AIS or risk of intracerebral bleeding^5^. Prior studies have shown an association of LA and poor outcome and intracerebral hemorrhage (ICH) after IVT^6^. Still the impact of LA on results after MT is a matter of debate. Smaller studies indicated an association of LA on clinical outcome while significant impact on rate of ICH couldn’t be seen^7,8^.

In contrast to pre-existing LA the blood pressure (BP) in acute phase of stroke is a modifiable risk factor with a special significance. High BP showed associations to poor clinical outcome, mortality and rate of sICH after stroke in prior studies^9^. However, the hemodynamic situation in ischemic penumbra differs between patients without reperfusion therapy and patients treated with IVT or with MT explained by reperfusion rates of 24% versus 46% versus 84%^10^. Nevertheless up to now formal recommendations of optimal blood pressure management after MT in acute phase of ischemic stroke are missing^11^.

We performed a retrospective study to proof the hypothesis that pre-existing LA and high BP as well as high BP variability in acute phase of stroke are associated with poor outcome, mortality and the occurrence of sICH after MT.

## Methods

### Study population

We retrospectively analyzed patients with AIS in anterior LVO treated with MT that were included in our local stroke registry between March 2009 and December 2016. A total of 531 patients were identified, of whom 521 patients were included in this study. In the excluded patients there were a lack of data (n = 2) or periinterventional LVO (n = 8). This study was approved by the local ethics committee of the medical faculty of Christian-Albrechts-University Kiel. All methods were carried out in accordance with relevant guidelines and regulations. Written informed consent was obtained from all patients.

### Patient characteristics

Information on age, sex, medical history, pre-existing conditions, laboratory measures, additional treatment with IVT or intraarterial lysis, stroke etiology using the TOAST classification^12^, imaging characteristics and interventional data were recorded (Table 1 for details). National Institutes of Health Stroke Scale (NIHSS) scores was assessed at the time of admission^13^.

**Table 1:** Baseline characteristics of the study population.

	n = 521
**Clinical data**	
Female gender	280 (53.7%)
Age, year	70.05 (± 13.72)
Admission NIHSS score	14 [10–18]
mRS at 3 months	3 [2–5]
Mortality	106 (21.5%)
Good clinical outcome (mRS90d 0–2)	202 (40.9%)
Good clinical short-term outcome (mRS on discharge 0–2)	177 (34%)
**Stroke mechanism**	
Cardioembolic	249 (47.8%)
Large artery atherosclerosis	95 (18.2%)
ESUS	122 (23.4%)
Competitive	29 (5.6%)
Other determined	26 (5%)
**Neuroradiological characteristics**	
ASPECTS score	8 [7–10]
**Occlusion site**	
Internal carotid artery	117 (22.5%)
Carotid-T	119 (22.8%)
Middle cerebral artery	368 (70.6%)
Hypoperfusion (Tmax > 6 s) (mL)	120.4 [75.4–172.3]
**Infarct core (CBF < 30%) (mL)**	
Mismatch volume (mL)	106.05 [65.8–147]
**Van Swieten Scale**	2 [1–4]
0	111 (22.3%)
1	83 (16.7%)
2	98 (19.7%)
3	73 (14.7%)
4	132 (26.6%)
Moderate–severe leukoaraiosis (vSS 2–4)	303 (61%)
**Treatment**	
Additional i.v. thrombolysis	226 (43.4%)
Additional i.a. thrombolysis	49 (9.4%)
Onset-to-thrombolysis time (min)	105 [80–130]
Onset-to-recanalization time (min)	275 [204–353]
Periinterventional stenting	113 (21.7%)
TICI ≥ 2b	439 (84.6%)
**Blood pressure**	
SBP admission	150 [130–167]
SBP preinterventional	160 [140–180]
SBP mean	135.4 [124.3]
SBP minimum	105 [92–120]
SBP maximum	168 [150–180]
SBP SD	17.5 [13.4–22.4]
DBP admission	81 [77–90]
DBP preinterventional	85 [75–95]
DBP mean	64.6 [58.1–70.1]
DBP minimum	54 [48–60]
DBP maximum	80 [70–87]
DBP SD	9.3 [7–11.9]
BP amplitude	70.9 [60.3–79]
**Laboratory data**	
Admission glucose (mg/dL)	119 [103–148]
International normalized ratio	1.0 [1–1.1]
Platelet count, 10^3^/µL	231 [187–274]
**Medical history**	
Atrial fibrillation	255 (51.8%)
Hyperlipidemia	259 (59.7%)
Coronary artery disease	113 (21.8%)
Hypertension	423 (81.8%)
Diabetes	114 (22%)
Prior stroke oder TIA	152 (29.2%)
**Pre-admission medications**	
Antiplatelets	187 (37%)
Vitamin K antagonists	46 (9.1%)
Clexane	18 (3.6%)
Noval oral anticoagulants	21 (4.2%)
Antihypertensive	362 (72%)
Antihyperglycemic	63 (12.5%)
Statin	126 (25%)
**Cerebral hemorrhage**	
0	339 (65.4%)
HI 1	35 (6.8%)
HI 2	84 (16.2%)
PH 1	27 (5.2%)
PH 2	33 (6.4%)
sICH	62 (11.9%)
aSICH	117 (22.6%)

Age is mean value with standard deviation, other data are median values with inter-quartile range in parentheses or numbers with percentages in parentheses.

*NIHSS* National Institutes of Health Stroke Scale, *mRS* modified Rankin Scale, *ESUS* Embolic Stroke Undetermined Source, *ASPECTS* Alberta Stroke Program Early CT Score, *CBF* cerebral blood flow, *vSS* Van Swieten Scale, *TICI* thrombolysis in cerebral infarction, *SBP* systolic blood pressure, *DBP* diastolic blood pressure, *TIA* transient ischemic attack, *HI* Hemorrhagic infarction, *PH* Parenchymal hematoma, *sICH* symptomatic intracerebral hemorrhage, *aSICH* ssymptomatic intracerebral hemorrhage.

BP was measured as initial BP in ambulance, pre- and periinterventional BP documented by anesthesia and postinterventional blood pressure was documented within the first 24 h after treatment. Mean BP and BP variability were obtained and calculated of 24-h BP measurement with extraction of blood pressure in 4-h-intervals. Maximum and minimum blood pressure was documented as highest and lowest documented blood pressure within the first 24 h after treatment. Further, the amplitude between mean diastolic BP (DBP) and systolic BP (SBP) was investigated. All BP values were collected separated in SBP and DBP parameters and mean arterial pressure (MBP) was calculated. BP variability is represented by the standard deviation (SD). There was a blood pressure control, which followed an in-house protocol. A mean systolic blood pressure of 140–180 mmHg was aimed for before the thrombectomy. After recanalization, normotensive blood pressure values were aimed for.

### Acute imaging and interventional therapy

All patients underwent non-contrast CT (NCCT) as well as extra- and intracranial CT angiography (CTA) on admission in emergency room. MT was performed by experienced neuroradiologists under general anesthesia. Successful recanalization was defined as achieving a Thrombolysis in Cerebral Infarction (TICI) score of 2b, 2c or 3^14^. The images were evaluated by two experienced neuroradiologists.

### Image analyses of leukoaraiosis

**Figure 1:**
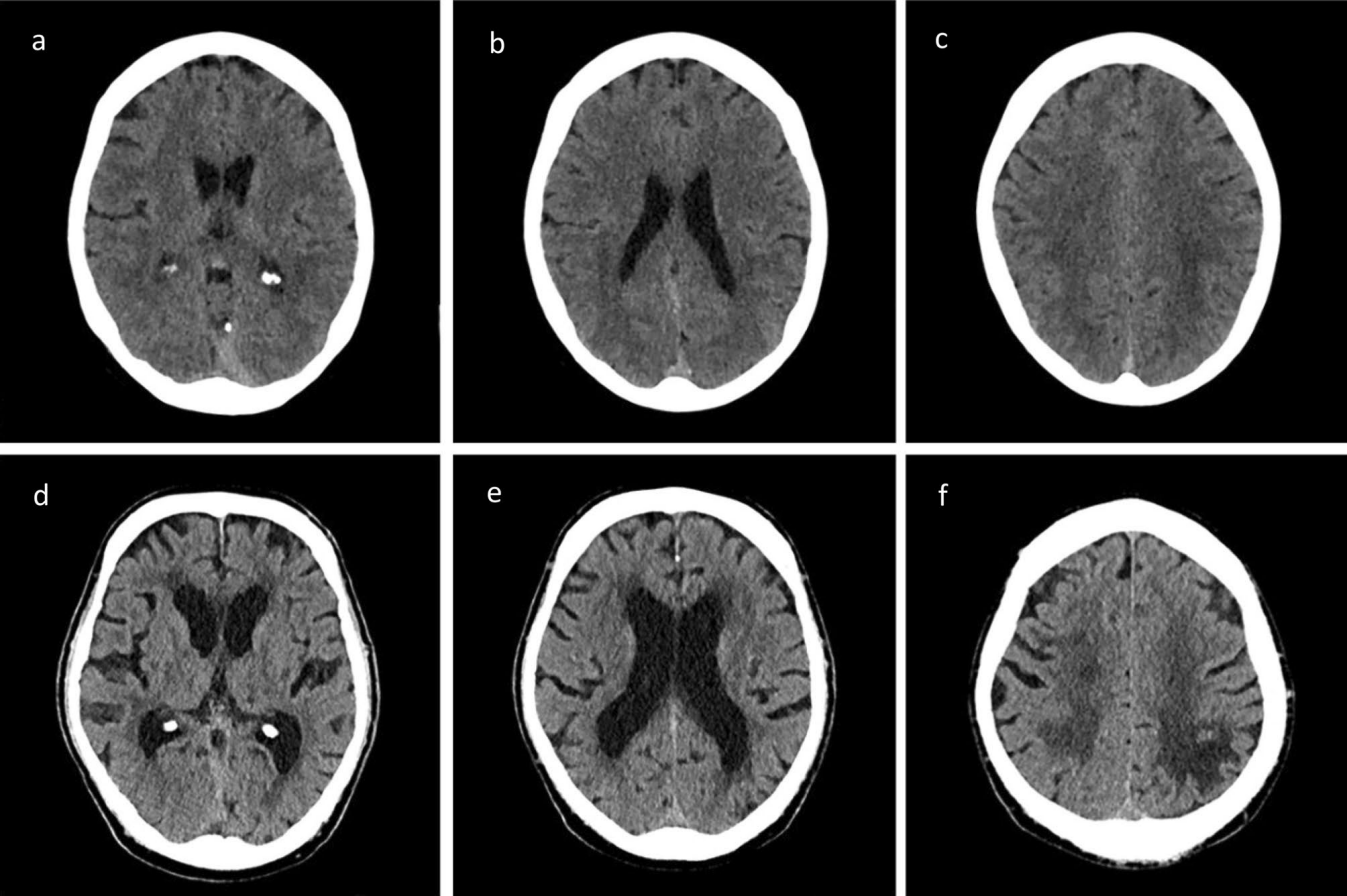
Assessment of the extent of leukoaraiosis in the NCCT using the van Swieten Scale (vSS). (**a**–**c**) Patient without leukoaraiosis, vSS 0; (**d**–**f**) patient with severe leukoaraiosis, vSS 4.

The grade of LA was assessed using the 4-point van Swieten scale (vSS)^15^ in NCCT on admission (Fig. 1). The grading was performed by an experienced neuroradiologist and trained rate rafter multi-staged training process by an experienced neuroradiologist. In case of different results in grading, a result was found by consent. Some patients were transferred secondary from an extern hospital to our clinic. In most of these cases the NCCT was repeated on admission. If the NCCT was not repeated due to time constraint, we used the external NCCTs to evaluate the grade of LA. In some patients with massive early ischemic changes in NCCT and large contralateral stroke in history it was not possible to assess the severity of LA. LA was dichotomized in absent-to-mild (vSS 0–1) versus moderate-to-severe (vSS2-4).

### Study outcomes

The modified Rankin Scale (mRS) was assessed 90 days after stroke by questionnaire or phone interview^16^. If the mRS90d was unavailable, we reconstructed the score from description in the rehabilitation report. A good clinical outcome was defined as mRS90d of 0–2, while a poor outcome was defined as mRS90d of 3–6. Mortality was assessed from mRS90d. SICH was classified according to ECASS-II-criteria, defined as any blood at any side in the brain on the NCCT causing a documented decrease in the NIHSS score of 4 points or more^17^.

### Statistics

Clinical and imaging baseline characteristics were compared regarding the parameter’s clinical outcome, mortality and sICH. Data are presented as number (percentage), median ± interquartile range (IQR) or mean ± SD according to its type of variable. For univariate and multivariate analysis, blood pressure was categorized in increments of 10 mmHg, standard deviation of blood pressure in increments of 5, age in increments of 10 years, and NIHSS in increments of 5 points. Between-group comparisons for categorical variables were made using the x^2^ test or in case of small expected frequencies, Fisher’s exact test. Metric and ordinal variables were compared using the Mann–Whitney U test. To identify independent predictors of each outcome parameter, multivariate binary logistic regression was performed including all variables with p < 0.05 in the univariable analyses. Associations are presented as OR with 95% CI. Results in the multivariate regression were considered statistically significant with p < 0.05. Statistical analyses were performed using IBM SPSS software (version 24).

## Results

### Study outcomes

Patient characteristics are shown in Table 1. We identified a total of 521 patients treated by MT during the study time with a mean age of 70 years (SD 13.7), including 280 females (53.7%). 226 patients (43.4%) underwent additional IVT and 49 patients (9.4%) additional intraarterial lysis. The median Alberta stroke program early CT score (ASPECTS) was 8 (IQR 7–10) and median NIHSS at admission was 14 (IQR 10–18). The occlusion site was internal carotid artery in 117 patients (22.5%), carotid-T in 119 patients (22.8%) and middle cerebral artery in 368 patients (70.6%). In several cases there was more than one occlusion site. Successful reperfusion with TICI ≥ 2b was reached in 439 patients (84.6%) with a median onset-to-recanalization-time of 275 min (IQR 204–353). At 3 months 202 patients (40.9%) achieved a good clinical outcome, while 106 patients (21.5%) died.

**Figure 2:**
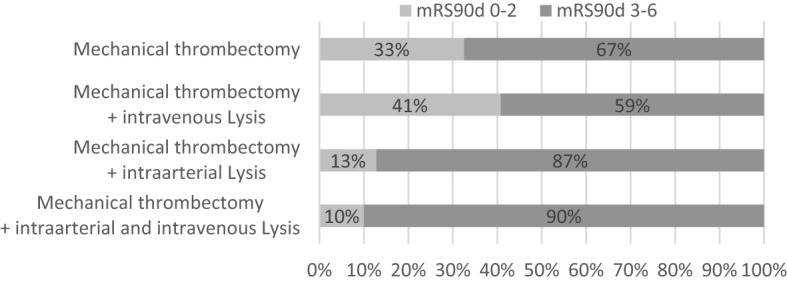
Clinical outcome in patients receiving mechanical thrombectomy with or without intravenous and intraarterial lysis. *mRS90d* modified Rankin Scale at 3 months.

Retrospectively, best clinical outcome was reached in patients receiving MT and additional IVT, while worst result was reached when getting MT and intraarterial lysis (IAT) or combined IAT and IVT (Fig. 2).

### Leukoaraiosis

There was no significant difference between patients with absent-to-mild versus moderate-to-severe LA regarding the time to IVT and recanalization, the early signs of infarction, occlusion location and the recanalization result.

**Figure 3:**
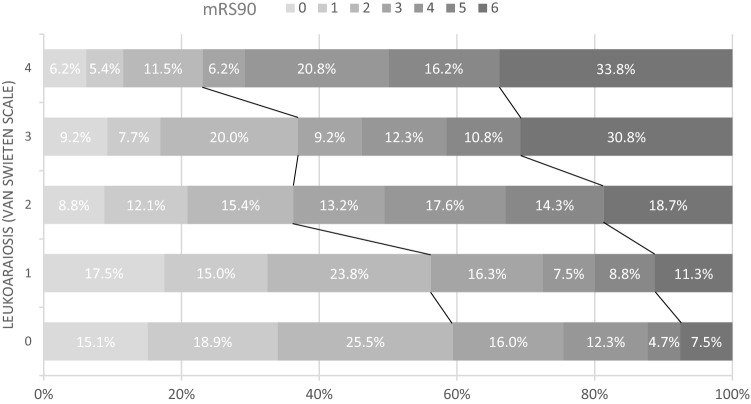
Clinical outcome in mRS90d depending on van Swieten Scale describing the degree of leukoaraiosis. *mRS90d* modified Rankin Scale at 3 months.

**Figure 4:**
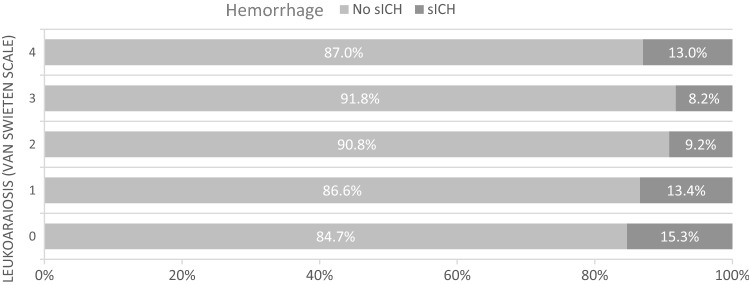
Occurrence of intracerebral hemorrhage depending on van Swieten Scale describing the degree of leukoaraiosis. *sICH *symptomatic intracerebral hemorrhage.

Independent predictors in multivariable logistic regression analyses of outcome parameters are shown in Table 2. LA was identified in 386 patients (77.7%), while 303 patients (61%) showed moderate-to-severe LA (vSS2-4). 69.6% of patients with moderate-to-severe LA reached a poor clinical outcome, compared to just 41.9% of patients absent-to-mild LA (Fig. 3). After adjusting for potential confounders, severity of LA was an independent predictor for a poor clinical outcome (OR 3.252; 95% CI 1.788–5.915; p < 0.001) and mortality (OR 3.189; 95% CI 1.35–7,531; p = 0.008). There was no different rate of sICH between patients with or without LA (14.5% vs. 10.6%, p = 0.193, Fig. 4). An additional IVT in patients with moderate-to-severe LA did not result in higher rates of sICH. Further the rate of IVT decreased with increasing vSS.

**Table 2:** Independent predictors of outcomes in multivariate logistic regression analysis.

Poor clinical outcome (mRS90d 3–6)			
	p value	OR	95% CI
Moderate–severe LA (vSS 2–4)	< 0.001	3.252	1.788–5.915
SBP maximum (per 10 mmHg increase)	0.009	1.208	1.048–1.393
sICH	0.009	8.672	1.730–43.474
Additional i.v. thrombolysis	0.016	0.497	0.281–0.879
TICI ≥ 2b	0.007	0.241	0.086–0.675
NIHSS (per 5 points increase)	< 0.001	1.649	1.268–2.146
Admission glucose (mg/dL)	0.021	1.009	1.001–1.016

Mortality			
Moderate–severe LA (vSS 2–4)	0.008	3.189	1.35–7.531
DBP mean (per 10 mm Hg increase)	< 0.001	0.534	0.379–0.752
sICH	< 0.001	4.9	2.037–11.791
TICI ≥ 2b	0.041	0.441	0.201–0.966
Age (per 10 years increase)	0.001	1.748	1.240–2.465
Vitamin K antagonists	0.006	3.409	1.410–8.241

sICH			
ASPECTS	0.001	0.734	0608–0.887
Cardioembolic cause	0.03	0.445	0.215–0.923

*mRS90d* modified Rankin Scale at 3 months, *sICH* symptomatic intracerebral hemorrhage, *LA* Leukoaraiosis, *vSS* van Swieten Scale, *SBP* systolic blood pressure, *DBP* diastolic blood pressure, *TICI* Thrombolysis in cerebral infarction, *NIHSS* National Institutes of Health Stroke Scale, *ASPECTS* Alberta stroke program early CT score.

### Blood pressure

There were no associations found between the initial BP in ambulance and the outcome parameters. Patients with a poor outcome had a significant higher mean SBP (136.4 vs. 132.9 mmHg, p = 0.026), maximum SBP (170 vs. 160 mmHg, p < 0.001) and amplitude between SBP and DBP (72.6 vs. 68 mmHg; p < 0.001) compared with those with good outcome. Moreover, the preinterventional SBP (160 vs. 157 mmHg; p = 0.04) and variability of SBP (18.4 vs. 16; p < 0.001) were higher in patients with poor outcome. Minimum DBP (50 vs. 55 mmHg; p = 0.016) was lower in patients with poor outcome. Only the association between the maximum SBP and clinical outcome remained after adjusting for pertinent covariates (OR 1.208 per 10 mmHg increase; 95% CI 1.048–1.393, p = 0.009).

Following BP parameters were associated with higher mortality: lower minimum SBP (100 vs. 105 mmHg, p = 0.003), higher variability of SBP (19.3 vs. 17; p = 0.007), lower mean and minimum DBP (60.6 vs. 65 mmHg; p < 0.001; 50 vs. 55 mmHg; p = 0.028) and lower mean MBP (83.6 vs. 88.3 mmHg; p < 0.001). In the multivariable analyses, low mean DBP remained associated with higher mortality (OR 0.534 per 10 mmHg increase; 95% CI 0.379–0.752; p < 0.001).

Only a higher variability of SBP (20 vs. 17.3 mmHg; p = 0.005) and higher variability of MBP (12.4 vs. 10.8 mmHg; p = 0.01) were associated with sICH, however after adjusting for covariates this association got lost.

### Further independent predictors for outcomes

Further independent predictors of a poor clinical outcome were a higher NIHSS on admission, higher admission glucose, additional IVT, futile recanalization and appearance of sICH (Table 2). Increased age, medication with vitamin K antagonists, futile recanalization and appearance of sICH were identified as further independent predictors of increased mortality. Independent predictors for the occurrence of sICH were a cardioembolic cause of the stroke and low ASPECTS.

Further details on the univariate analyzes of the endpoints clinical outcome, mortality and sICH can be found in the supplementary information (Supplementary Tables 1–3).

## Discussion

MT is a therapeutic method for AIS patients with LVO that has been established since 2015^1^. Symptomatic intracerebral hemorrhage as a possible complication of AIS is associated with poor results^18^. LA as a radiologically visible pre-existing microangiopathic damage of white matter, as well as the course of blood pressure in the acute phase of a stroke, are both controversial discussed factors for poor outcome especially in endovascular treated patients. Since LA is directly caused by hypertension, it is not yet clear if blood pressure or LA correspond more significantly to clinical outcome in patients with LA. Further, blood pressure is a modifiable therapeutic target. Optimal blood pressure limits might therefore help to enhance good clinical outcome in large vessel occlusion stroke.

Clinical outcome was best in group of patients receiving additional IVT, while the group of patients getting IAT or combined IVT and IAT had worst results. In the multivariate analysis, the only significant effect remained a positive effect of additional intravenous lysis on the clinical outcome. One of the reasons for these differences is probably the shorter onset to recanalization time in IVT patients and individual attempts at healing using IAT in patients with unsuccessful mechanical recanalization.

### Leukoaraiosis

Our study showed an independent association between LA and poor clinical outcome as well as mortality, while no association could be found between LA and the rate of sICH. Gou et al.^7^ confirmed a severe LA as an independent predictor of a poor clinical result and increased mortality as well as futile recanalization, defined as a poor clinical result despite successful recanalization. An association between severe LA and the occurrence of sICH could not be shown. The rate of good clinical outcome (45.5%), mortality (23.9%) and sICH (10.4%) was similar to the results of our study. Numerous other studies were able to confirm LA as an independent risk factor for an poor clinical result^19,20^ and increased mortality^21,22^ while few studies were not able to demonstrate an independent influence on the clinical result^23^ or mortality^24^. Meta-analyses have shown an association between severe or moderate to severe LA and an poor clinical result and higher mortality^25,26^. The largest meta-analysis so far was able to confirm the association between LA and the clinical result, but not the association with mortality after 90 days^27^. In a first secondary evaluation of the randomized MR CLEAN thrombectomy study, despite the influence of LA on the clinical outcome, no effect on the success of a thrombectomy could be found, so that patients with LA seem to benefit from thrombectomy as well^28^. In summary, the results of this study are consistent to majority of other studies, but with larger collective than in other studies and additional involved blood pressure values.

Regarding the occurrence of sICH, other smaller studies^8,20^ as well as meta-analyses^25,27^ were also unable to identify any association between the extent of LA and the rate of sICH. In opposite Rastogi et al. demonstrated in their meta-analysis a significant association between LA and the rate of sICH^26^. However, they included not only patients treated with thrombectomy in the meta-analysis, but also patients treated purely with IVT. Subgroup analyses were able to limit the significant association of LA with the bleeding rate after IVT, but not after MT (with and without additional IVT). Our results of missing association between LA and sICH are therefore consistent with other studies.

The association between LA with the clinical outcome after stroke could be explained by a reduced possibility of compensation and reduced ischemia tolerance in the acute phase of a vascular occlusion with a reduced microangiopathic reserve capacity and thus a larger infarct volume^29^. Zhang et al. showed that LA is independently associated with the infarct volume and that both the LA and the infarct volume correlate independently with the clinical result. They also advocated the hypothesis that the reduced ability to compensate the neural structures results in reduced cerebral plasticity^29^. The identification of LA as an independent risk factor for poor collateral status^30^ supports the hypothesis about the pathomechanism of cerebrovascular dysfunction.

With respect of the current literature and our study results, LA seems to be independently associated with a poor clinical outcome and increased mortality after MT, but not with the occurrence of sICH. However, despite these consistent results, it must be taken into account that there is also a significant association between the degree of LA and other relevant cardiovascular comorbidities such as hypertension, atrial fibrillation, previous strokes and coronary heart disease^25^ and the patient's age^31–33^, which are partial associated with the treatment outcome^34^. In addition, it can be assumed that, considering the multimorbidity associated with LA, the pre-stroke MRS could be also higher in patients with LA. Therefore, even with good statistical methods, the influence of LA on the treatment result cannot be clarified completely independently of other influencing factors at the current point in time.

### Blood pressure

In this study, an increased maximum SBP within the first 24 h after MT was an independent predictor of a poor clinical outcome and a decreased mean DBP an independent predictor of increased mortality. However, there was no independent association between the numerous BP values and the rate of sICH. Pre-existing hypertension was not an independent risk factor.

In recent years, numerous studies have been published on the influence of BP in the acute phase after MT. The BP is an important factor of influence due to its modifiable character. Nevertheless the optimal goals of BP management have not yet been adequately investigated^11,35^.

In previous studies of patients treated by MT, an influence of the SBP on admission on the clinical result could be shown: Mulder et al. were able to show a U-shaped correlation to the clinical result in a secondary evaluation of the randomized MR CLEAN^36^, while other studies identified a high SBP pressure as an independent predictor of a poor clinical result^37,38^. A J- or U-shaped relationship to the SBP with an optimum of 120 and 157 mmHg was shown for mortality^36,38^. However, the MR CLEAN study could not show any correlation between BP and the effect of the MT^36^. Other thrombectomy studies as well as our study, examined various parameters of BP within the first 24 h. Especially high maximum SBP seem to be independently associated with a poor clinical outcome and increased mortality^39–41^. A large BP variability as an independent risk factor for a poor clinical outcome^42–44^ and an increased mortality^44^ could be observed. Cho et al. also demonstrated a negative effect of increased mean SBP and observed an increased influence of these parameters in successfully recanalized patients compared to non-recanalized patients^44^. Two meta-analysis described an association between the variability of the SBP and a poor clinical result^45,46^, but no association with the 90-day mortality^46^. Further meta-analyses confirmed a significant association of increased SBP after MT^47^ or pre- and post-interventional^9^ with a poor clinical result. In addition, increased post-interventional systolic and diastolic BP values were associated with increased mortality^9^. Although in the present work only a high maximum systolic blood pressure or low mean diastolic blood pressure showed an independent association with the clinical result or mortality, in summary, high blood pressure and high blood pressure variability appear to be associated with an unfavorable clinical result and an increased one to be associated with mortality.

A possible explanation for the influence of BP on the development of sICH is that the infarct core and penumbra are particularly at risk for reperfusion damage after restoration of blood flow due to increased vascular permeability^48^. The increased permeability of the vessels is based on cellular and molecular mechanisms in ischemic tissue, for example the formation of reactive radicals and the release of proinflammatory cytokines^48^. A disruption of the cerebral autoregulation as a further consequence of the reperfusion damage leads in turn to a reduced control over the cerebral blood flow and an increased susceptibility to high blood pressures. This contributes to further damage through increased perfusion pressure^48,49^.

This study has some strengths and limitations. Compared with other retrospective studies, this study includes with a cohort of 521 patients one of the largest cohorts. We also included lots of relevant parameters in the multivariate analyses. The study was limited by its retrospective design and data from a single center. Second, the grading of the LA with the vSS based on CT images alone is somewhat unprecise and estimated, however more accurate data from MRI are difficult to achieve in such a large cohort.

The results of this study provide important information on the relationship BP, LA and the clinical result or the risk of bleeding after MT. The results require further confirmation by prospective, randomized and controlled studies or secondary evaluations of the randomized thrombectomy studies with regard to the course of BP and LA. In summary, however, at the present time it can be stated that even a severe LA is not a contraindication for MT and that it is obviously not only the absolute BP values that are the decisive risk factor for sICH after MT, but that the range between systolic and diastolic BP and high SBP are decisive risk parameters for secondary hemorrhage.

## Conclusion

In conclusion, our study provides previous data that the severity of LA is independently associated with poor 90-day outcome and mortality in AIS patients undergoing MT. Still these results shouldn’t lead to withhold MT. A lower BP variability and avoidance of SBP peaks and low DBP may be protecting factors for clinical outcome, mortality and rate of sICH. However, these observations require validation by larger prospective and multicenter studies.

## Supplementary Information

Supplementary Information.

## Data Availability

All data generated or analyzed during this study are included in this published article (and its Supplementary Information files).
