# Supplementary material for: Impact of leukoaraiosis or blood pressure on clinical outcome, mortality and symptomatic intracerebral hemorrhage after mechanical thrombectomy in acute ischemic stroke
Source: Sci Rep. 2022 Dec 16;12:21750. doi: 10.1038/s41598-022-25171-9 (PMC9758212; doi:10.1038/s41598-022-25171-9)
Supplement: Supplementary file 1 — Supplementary Information. [file 41598_2022_25171_MOESM1_ESM.docx]

**Supplementary information**

Supplementary information table 1: Differences in patient clinical data between patients with good and poor clinical outcomes using the mRS90d. Univariate analysis specifying p-values

|  | Good clinical outcome (n=202) | Poor clinical outcome (n=292) | p-value |
| --- | --- | --- | --- |
| Clinical data |  |  |  |
| Female gender n (%) | 100 (49,5%) | 169 (57,9%) | 0,066^b^ |
| Age, years (mean ± SD) | 65,3 ± 14,4 | 73,5 ± 12,4 | **<0,001^a^** |
| Admission NIHSS score median [IQR] | 12 [8-16] | 16 [12-20] | **<0,001^a^** |
| sICH n (%) | 5 (2,5%) | 54 (18,6%) | **<0,001^b^** |
| Stroke mechanism n (%) |  |  |  |
| Cardioembolic | 91 (45%) | 139 (47,6%) | 0,576^b^ |
| Large artery atherosclerosis | 40 (19,8%) | 54 (18,5%) | 0,716^b^ |
| ESUS | 43 (21,3%) | 73 (25%) | 0,339^b^ |
| Neuroradiological characteristics |  |  |  |
| ASPECTS score median [IQR] | 9 [7-10] | 8 [7-10] | **0,002^a^** |
| Occlusion site n (%) |  |  |  |
| Internal carotid artery | 48 (23,8%) | 64 (21,9%) | 0,63^b^ |
| Carotid-T | 42 (20,8%) | 71 (24,3%) | 0,359^b^ |
| Middle cerebral artery | 144 (71,3%) | 204 (69,9%) | 0,733^b^ |
| Hypoperfusion (Tmax>6s) (ml) median [IQR] | 109,3 [65,8-160,3] | 132 [79,5-188,9] | **0,016^a^** |
| Infarct core (CBF <30%) (ml) median [IQR] | 5 [0-15] | 6 [0-21] | 0,214^a^ |
| Mismatch volume (ml) median [IQR] | 101 [57,8-143,3] | 111 [69,5-156,5] | 0,073^a^ |
| Moderate–severe leukoaraiosis  (vSS 2–4) n (%) | 87 (55,4%) | 199 (71,8%) | **<0,001^b^** |
| Treatment |  |  |  |
| Additional i.v. thrombolysis n (%) | 104 (51,5%) | 112 (38,4%) | **0,004^b^** |
| Additional i.a. thrombolysis n (%) | 8 (4%) | 36 (12,3%) | **0,001^b^** |
| Onset-to-thrombolysis time (min) median [IQR] | 95 [73-120] | 115 [83-140,5] | **0,044^a^** |
| Onset-to-recanalization time (min) median [IQR] | 244,5 [188,3-349,5] | 289 [227,3-355,5] | **0,015^a^** |
| Periinterventional stenting n (%) | 54 (26,7%) | 58 (19,9%) | 0,073^b^ |
| TICI ≥ 2b n (%) | 190 (94,1%) | 228 (78,4%) | **<0,001^b^** |
| Blood pressure median [IQR] |  |  |  |
| SBP Admission | 140 [130-160] | 150 [130-170] | 0,066^a^ |
| SBP Preinterventional | 157 [139-179] | 160 [140-184,5] | **0,04^a^** |
| SBP Mean | 132,9 [123,9-141,7] | 136,4 [123,8-146,4] | **0,026^a^** |
| SBP Minimum | 105 [95-120] | 103 [90-120] | 0,246^a^ |
| SBP Maximum | 160 [150-180] | 170 [160-185] | **<0,001^a^** |
| SBP SD | 16 [12,2-20,9] | 18,4 [14,2-23,3] | **<0,001^a^** |
| DBP Admission | 85 [78-90,5] | 80 [76-90] | 0,597^a^ |
| DBP Preinterventional | 85 [75-95] | 85 [75-95] | 0,279^a^ |
| DBP Mean | 65 [59-70,7] | 64 [57,4-69,9] | 0,22^a^ |
| DBP Minimum | 55 [50-60] | 50 [45-60] | **0,016^a^** |
| DBP Maximum | 78 [70-85] | 80 [70-88] | 0,362^a^ |
| DBP SD | 9,5 [6,8-11,9] | 9,2 [7,2-11,8] | 0,89^a^ |
| MAP Admission | 106 [94,9-114,4] | 105,2 [94,1-116,7] | 0,967^a^ |
| MAP Preinterventional | 106,7 [98,3-120] | 110 [98,3-123,3] | 0,168^a^ |
| MAP Mean | 87,6 [82,2-93,1] | 87,7 [80,4-95,5] | 0,847^a^ |
| MAP SD | 10,7 [8-13] | 11,4 [8,7-14,7] | 0,122^a^ |
| BP Amplitude | 68 [57,9-76,1] | 72,6 [62,5-79,9] | **0,001** |
| Laboratory data median [IQR] |  |  |  |
| Admission glucose (mg/dL) | 112 [101-135] | 125 [105-159] | **<0,001^a^** |
| International normalized ratio | 1,03 [0,99-1,09] | 1,05 [1-1,13] | **0,009^a^** |
| Platelet count, 10³/µL | 227 [183-263,8] | 235 [190,5-282] | 0,148^a^ |
| Medical history n (%) |  |  |  |
| Atrial fibrillation | 83 (42,1%) | 153 (56,9%) | **0,002^b^** |
| Hyperlipidemia | 106 (59,9%) | 135 (57,9%) | 0,692^b^ |
| Coronary artery disease | 30 (14,9%) | 77 (26,6%) | **0,002^b^** |
| Hypertension | 141 (70,1%) | 258 (89,3) | **<0,001^b^** |
| Diabetes | 32 (15,9%) | 77 (26,6%) | **0,005^b^** |
| Prior stroke or TIA | 47 (23,3%) | 101 (34,6%) | **0,007^b^** |
| Pre-admission medications n (%) |  |  |  |
| Antiplatelets | 63 (32%) | 116 (41%) | **0,045^b^** |
| Vitamin K antagonists | 9 (4,5%) | 34 (12,2%) | **0,004^b^** |
| Clexane | 1 (0,5%) | 13 (4,6%) | **0,008^b^** |
| Noval oral anticoagulants | 6 (3%) | 14 (5%) | 0,289^b^ |
| Antihypertensive | 123 (62,4%) | 220 (78,6%) | **<0,001^b^** |
| Antihyperglycemic | 19 (9,6%) | 42 (15%) | 0,085^b^ |
| Statin | 36 (18,3%) | 80 (28,6%) | **0,01^b^** |

*NIHSS* National Institutes of Health Stroke Scale, *sICH* symptomatic intracerebral hemorrhage, *ESUS* Embolic Stroke Undetermined Source, *ASPECTS* Alberta Stroke Program Early CT Score, *CBF* cerebral blood flow, vSS Van Swieten Scale, *TICI* thrombolysis in cerebral infarction, SBP systolic blood pressure, DBP diastolic blood pressure, *MAP* mean arterial pressure, *BP* blood pressure, *TIA* transient ischemic attack

^a^Mann-Whitney-U-Test, ^b^Chi-Quadrat-Test

Supplementary information table 2: Differences in patient data between surviving and deceased patients after 3 months. Univariate analysis specifying p-values.

|  | Survive (n=388) | Death (n=106) | p-value |
| --- | --- | --- | --- |
| Clinical data |  |  |  |
| Female gender n (%) | 207 (53,4%) | 62 (58,5%) | 0,346^b^ |
| Age, years (mean ± SD) | 68,1 ± 14 | 77,5 ± 10,6 | **<0,001^a^** |
| Admission NIHSS score median [IQR] | 14 [10-18] | 16 [12-21] | **<0,001^a^** |
| sICH n (%) | 27 (7%) | 32 (30,5%) | **<0,001^b^** |
| Stroke mechanism n (%) |  |  |  |
| Cardioembolic | 176 (45,4%) | 54 (50,9%) | 0,307^b^ |
| Large artery atherosclerosis | 81 (20,9%) | 13 (12,3%) | **0,045^b^** |
| ESUS | 87 (22,4%) | 29 (27,4%) | 0,288^b^ |
| Neuroradiological characteristics |  |  |  |
| ASPECTS score median [IQR] | 8 [7-10] | 9 [7-10] | 0,162^a^ |
| Occlusion site n (%) |  |  |  |
| Internal carotid artery | 87 (22,4%) | 25 (23,6%) | 0,8^b^ |
| Carotid-T | 93 (24%) | 20 (18,9%) | 0,268^b^ |
| Middle cerebral artery | 270 (69,6%) | 78 (73,6%) | 0,424^b^ |
| Hypoperfusion (Tmax>6s) (ml) median [IQR] | 118 [70-167,3 | 132 [87,9-193,7] | 0,067^a^ |
| Infarct core (CBF <30%) (ml) median [IQR] | 5,3 [0-18] | 5 [0-18] | 0,859^a^ |
| Mismatch volume (ml) median [IQR] | 104,6 [61,8-146] | 112 [72-173] | 0,095^a^ |
| Moderate–severe leukoaraiosis  (vSS 2–4) n (%) | 205 (54,8%) | 81 (82,7%) | **<0,001^b^** |
| Treatment |  |  |  |
| Additional i.v. thrombolysis n (%) | 181 (46,6%) | 35 (33%) | **0,012^b^** |
| Additional i.a. thrombolysis n (%) | 32 (8,2%) | 12 (11,3%) | 0,325^b^ |
| Onset-to-thrombolysis time (min) median [IQR] | 100 [80-130] | 120 [77,5-142,5] | 0,658^a^ |
| Onset-to-recanalization time (min) median [IQR] | 275 [206-352,5] | 280 [200-354,5] | 0,897^a^ |
| Periinterventional stenting n (%) | 94 (24,2%) | 18 (17%) | 0,114^b^ |
| TICI ≥ 2b n (%)TICI ≥ 2b n (%) | 341 (87,9%) | 77 (73,3%) | **<0,001^b^** |
| Blood pressure median [IQR] |  |  |  |
| SBP Admission | 149 [130-166] | 151 [127,3-170] | 0,446^a^ |
| SBP Preinterventional | 160 [140-180] | 161 [138-190] | 0,464^a^ |
| SBP Mean | 135,7 [124,7-145] | 134,2 [119,4-142,6] | 0,21^a^ |
| SBP Minimum | 105 [95-120] | 100 [90-110] | **0,003^a^** |
| SBP Maximum | 165 [150-180] | 170 [150-185] | 0,623^a^ |
| SBP SD | 17 [13-22,2] | 19,3 [15,2-23,9] | **0,007^a^** |
| DBP Admission | 83 [78-90] | 80 [70-90] | 0,223^a^ |
| DBP Preinterventional | 85 [75-95] | 85 [75-95] | 0,91^a^ |
| DBP Mean | 65 [59,2-70,7] | 60,6 [56,1-66,3] | **<0,001^a^** |
| DBP Minimum | 55 [60-50] | 50 [45-60] | **0,028^a^** |
| DBP Maximum | 80 [70-88] | 75 [65-85] | 0,111^a^ |
| DBP SD | 9,5 [6,9-11,9] | 9 [7,2-11,7] | 0,864^a^ |
| MAP Admission | 106,7 [96,3-115,2] | 103,3 [90-120] | 0,721^a^ |
| MAP Preinterventional | 108,3 [98,3-121,1] | 111,7 [95-124] | 0,728^a^ |
| MAP Mean | 88,3 [82,6-95,1] | 83,6 [78,7-92,5] | **<0,001^a^** |
| BP SD | 10,8 [8,2-13,8] | 11,9 [8,8-11,9] | 0,137^a^ |
| BP amplitude | 69,9 [59,7-78,6] | 72,6 [61,4-80,2] | 0,252 |
| Laboratory data median [IQR] |  |  |  |
| Admission glucose (mg/dL) | 117 [102-145,5] | 126 [108-157] | **0,015^a^** |
| International normalized ratio | 1,03 [1-1,1] | 1,06 [0,99-1,2] | 0,073^a^ |
| Platelet count, 10³/µL | 230 [182,8-269] | 241 [197-291] | 0,053^a^ |
| Medical history n (%) |  |  |  |
| Atrial fibrillation | 173 (45,6%) | 63 (72,4%) | **<0,001^b^** |
| Hyperlipidemia | 188 (57,5%) | 53 (63,9%) | 0,293^b^ |
| Coronary artery disease | 68 (17,6%) | 39 (37,5%) | **<0,001^b^** |
| Hypertension | 303 (78,5%) | 96 (92,3%) | **0,001^b^** |
| Diabetes | 82 (21,2%) | 27 (26%) | 0,298^b^ |
| Prior stroke or TIA | 101 (26%) | 47 (44,3%) | **<0,001^b^** |
| Pre-admission medications n (%) |  |  |  |
| Antiplatelets | 132 (35,1%) | 47 (45,2%) | 0,06^b^ |
| Vitamin K antagonists | 26 (7%) | 17 (16,3%) | **0,003^b^** |
| Clexane | 11 (2,9%) | 3 (2,9%) | 1^c^ |
| Noval oral anticoagulants | 13 (3,5%) | 7 (6,7%) | 0,165^c^ |
| Antihypertensive | 258 (69,2%) | 85 (81,7%) | **0,012^b^** |
| Antihyperglycemic | 45 (12,1%) | 16 (15,4%) | 0,37^b^ |
| Statin | 80 (21,4%) | 36 (34,6%) | **0,006^b^** |

*NIHSS* National Institutes of Health Stroke Scale, *sICH* symptomatic intracerebral hemorrhage, *ESUS* Embolic Stroke Undetermined Source, *ASPECTS* Alberta Stroke Program Early CT Score, *CBF* cerebral blood flow, vSS Van Swieten Scale, *TICI* thrombolysis in cerebral infarction, SBP systolic blood pressure, DBP diastolic blood pressure, *MAP* mean arterial pressure, *BP* blood pressure, *TIA* transient ischemic attack

^a^Mann-Whitney-U-Test, ^b^Chi-Quadrat-Test, ^c^Fisher´s exact Test

Supplementary information table 3: Differences in clinical patient data between patients with and without occurrence of SICH. Univariate analysis specifying p-values.

|  | No sICH (n=457) | sICH (n=62) | p-value |
| --- | --- | --- | --- |
| Clinical data |  |  |  |
| Female gender n (%) | 244 (53,4%) | 35 (56,5%) | 0,65^b^ |
| Age, years (mean ± SD) | 70,3 ± 13,8 | 67,8 ± 12,8 | 0,182^a^ |
| Admission NIHSS score median [IQR] | 14 [10-18] | 15,5 [13,75-20] | **0,001^a^** |
| Stroke mechanism n (%) |  |  |  |
| Cardioembolic | 226 (49,5%) | 22 (35,5%) | **0,039^b^** |
| Large artery atherosclerosis | 85 (18,6%) | 9 (14,5%) | 0,433^b^ |
| ESUS | 100 21,9(%) | 22 (35,5%) | **0,018^b^** |
| Neuroradiological characteristics |  |  |  |
| ASPECTS score median [IQR] | 8 [7-10] | 7 [6.9] | **0,013^a^** |
| Occlusion site n (%) |  |  |  |
| Internal carotid artery | 100 (21,9%) | 16 (25,8%) | 0,486^b^ |
| Carotid-T | 102 (22,3%) | 17 (27,4%) | 0,37^b^ |
| Middle cerebral artery | 318 (69,6%) | 49 (79%) | 0,125^b^ |
| Hypoperfusion (Tmax>6s) (ml) median [IQR] | 118 [73,7-167,3] | 152 [94-211] | **0,02^a^** |
| Infarct core (CBF <30%) (ml) median [IQR] | 5 [0-17] | 11 [3-44] | **0,005^a^** |
| Mismatch volume (ml) median [IQR] | 105,5 [63-146] | 117 [76-168] | 0,113^a^ |
| Moderate–severe leukoaraiosis  (vSS 2–4) n (%) | 270 (62,1%) | 32 (53,3%) | 0,193^b^ |
| Treatment |  |  |  |
| Additional i.v. thrombolysis n (%) | 201 (44%) | 25 (40,3%) | 0,585^b^ |
| Additional i.a. thrombolysis n (%) | 43 (9,4%) | 6 (9,7%) | 0,946^b^ |
| Onset-to-thrombolysis time (min) median [IQR] | 100,5 [80-130] | 110 [81,5-130,5] | 0,709^a^ |
| Onset-to-recanalization time (min) median [IQR] | 275 [201,8-356] | 273,5 [231,5-338,3] | 0,965^a^ |
| Periinterventional stenting n (%) | 95 (20,8%) | 18 (29%) | 0,14^b^ |
| TICI ≥ 2b n (%) | 394 (86,4%) | 44 (72,1%) | **0,004^b^** |
| Blood pressure median [IQR] |  |  |  |
| SBP Admission | 150 [130-167] | 150 [130-168] | 0,841^a^ |
| SBP Preinterventional | 160 [140-180] | 157,5 [140-170] | 0,287^a^ |
| SBP Mean | 135,6 [124,7-145,8] | 132,1 [121-141,4] | 0,109^a^ |
| SBP Minimum | 105 [94,25-120] | 100 [90-110] | 0,067^a^ |
| SBP Maximum | 167 [150,5-184] | 170 [150-180] | 0,771^a^ |
| SBP SD | 17,3 [13,2-22,1] | 20 [15,6-24] | **0,005^a^** |
| DBP Admission | 81,5 [77-90] | 80 [80-96,8] | 0,776^a^ |
| DBP Preinterventional | 85 [75-95] | 81 [75-90] | 0,176^a^ |
| DBP Mean | 64,7 [58-70,4] | 62,8 [58,1-70] | 0,625^a^ |
| DBP Minimum | 55 [48-60] | 50 [45-60] | 0,533^a^ |
| DBP Maximum | 80 [70-85] | 80 [70-89] | 0,574^a^ |
| DBP SD | 9,4 [6,9-11,8] | 9,1 [7,4-12,8] | 0,346^a^ |
| MAD Admission | 106,3 [95,3-116] | 105,8 [96,4-117,4] | 0,815^a^ |
| MAD Preinterventional | 109,7 [98,3-122,5] | 106,7 [96,7-113,3] | 0,117^a^ |
| MAD Mean | 88,1 [82-94,9] | 85 [79,5-93,7] | 0,128^a^ |
| MAD SD | 10,8 [8,2-13,8] | 12,4 [9,4-15,7] | **0,01^a^** |
| BP Amplitude | 71,2 [60,4-79,3] | 69,3 [57,3-75,3] | 0,103 |
| Laboratory data median [IQR] |  |  |  |
| Admission glucose (mg/dL) | 117,5 [102,3-146] | 127 [108-163] | **0,041^a^** |
| International normalized ratio | 1,04 [1-1,1] | 1,05 [1-1,18] | 0,266^a^ |
| Platelet count, 10³/µL | 230 [186-273,3] | 244 [192-286] | 0,525^a^ |
| Medical history n (%) |  |  |  |
| Atrial fibrillation | 227 (50,9%) | 27 (60%) | 0,244^b^ |
| Hyperlipidemia | 237 (60%) | 21 (55,3%) | 0,57^b^ |
| Coronary artery disease | 96 (21%) | 17 (28,3%) | 0,197^b^ |
| Hypertension | 367 (80,7%) | 55 (90,2%) | 0,071^b^ |
| Diabetes | 98 (21,4%) | 16 (26,7%) | 0,359^b^ |
| Prior stroke or TIA | 135 (29,5%) | 17 (27,4%) | 0,731^b^ |
| Pre-admission medications n (%) |  |  |  |
| Antiplatelets | 163 (36,5%) | 24 (41,4%) | 0,466^b^ |
| Vitamin K antagonists | 38 (8,5%) | 8 (14%) | 0,176^b^ |
| Clexane | 16 (3,6%) | 2 (3,5%) | 1^c^ |
| Noval oral anticoagulants | 20 (4,5%) | 1 (1,8%) | 0,494^c^ |
| Antihypertensive | 317 (71,2%) | 44 (77,2%) | 0,346^b^ |
| Antihyperglycemic | 54 (12,1%) | 9 (15,8%) | 0,433^b^ |
| Statin | 114 (25,6%) | 11 (19,3%) | 0,299^b^ |

*sICH* symptomatic intracerebral hemorrhage, *NIHSS* National Institutes of Health Stroke Scale, *ESUS* Embolic Stroke Undetermined Source, *ASPECTS* Alberta Stroke Program Early CT Score, *CBF* cerebral blood flow, vSS Van Swieten Scale, *TICI* thrombolysis in cerebral infarction, SBP systolic blood pressure, DBP diastolic blood pressure, *MAP* mean arterial pressure, *BP* blood pressure, *TIA* transient ischemic attack

^a^Mann-Whitney-U-test, ^b^Chi-Quadrat-test, ^c^Fisher´s exact test
